# Rotavirus genotypes associated with childhood severe acute diarrhoea in southern Ghana: a cross-sectional study

**DOI:** 10.1186/1743-422X-10-287

**Published:** 2013-09-14

**Authors:** Christabel C Enweronu-Laryea, Kwamena W Sagoe, Susan Damanka, Belinda Lartey, George E Armah

**Affiliations:** 1 Department of Child Health, University of Ghana Medical School, College of Health Sciences, University of Ghana, Accra, Ghana; 2 Department of Microbiology, University of Ghana Medical School, College of Health Sciences, University of Ghana, Accra, Ghana; 3 Noguchi Memorial Institute of Medical Research, College of Health Sciences, University of Ghana, Accra, Ghana

**Keywords:** Rotavirus, Gastroenteritis, Diarrhoea, Genotypes, Ghana, Immunization

## Abstract

**Background:**

Rotavirus immunization has been effective in developed countries where genotype G1P[8] is the predominant rotavirus strain. Knowledge of circulating strains in a population before introduction of rotavirus immunization program will be useful in evaluating the effect of the intervention.

**Methods:**

Rotavirus was identified by enzyme immuno-assay (EIA) on stool specimens of children (age 0 – 59 months) hospitalized with acute gastroenteritis from August 2007 to February 2011 in Accra, Ghana. Rotavirus positive specimens were further characterized by polyacrylamide gel electrophoresis (PAGE) and reverse-transcriptase polymerase chain reaction (RT-PCR).

**Results:**

Of the 2277 acute gastroenteritis hospitalizations 1099 (48.2%) were rotavirus-positive by EIA. Of the 1099 cases 977 (89%) were PAGE positive. All EIA positive specimens were further subjected to RT-PCR and 876 (79.7%) had sufficient material for characterization. Of these 876 cases, 741 (84.6%) were assigned G genotype, 709 (80.9%) P genotype, and 624 (71.2%) both G and P genotypes. We identified 8 G genotypes (G1, G2, G3, G4, G8, G9, G10, G12) and 3 P genotypes (P[4], P[6], P[8]). G1 (50.9%), G2 (18.8%), G3 (12.8%), P[8] (36.1%) and P[6] (30.7%) were the most prevalent. The most prevalent genotype combination was G1P[8] (28%). Mixed G (7.3%) and P (24.2%) genotypes were not uncommon. There was year-by-year and seasonal variations for most genotypes.

**Conclusion:**

There is great diversity of rotavirus strains in children with severe gastroenteritis in southern Ghana. Even though cross-protection with vaccine-induced immunity occurs, continued strain surveillance is recommended after the introduction of rotavirus vaccine in the national immunization program.

## Background

Rotavirus gastroenteritis significantly contributes to childhood morbidity and mortality in developing countries [1,2]. The double-stranded RNA virus is classified into 8 groups (A – H) based on antigenicity and nucleotide sequence identities of the VP6 gene [3,4]. The two outer capsid proteins VP4 (P type specificity) and VP7 (G type specificity) stimulate human immunological response and are targets for the development of rotavirus vaccines [5].

Currently, 27 G and 35 P genotypes have been identified in humans and animals [6]. Global epidemiologic surveys have identified G1P[8], G2P[4], G3P[8], G4P[8], and G9P[8] as the most common circulating strains associated with diarrhoea in humans [7]. However, recent studies in developing countries have shown greater diversity and this may impact the efficacy of rotavirus vaccination programs in these countries [8-12].

The World Health Organization (WHO) recommends surveillance for the burden of rotavirus disease and circulating rotavirus strains, before and after inclusion of rotavirus vaccination in national expanded programs on immunization [13]. Previous work has been published on rotavirus strains in the dry savannah northern part of Ghana but little is known about circulating rotavirus strains in the warm humid southern part of the country. This report presents the rotavirus genotypes identified in children hospitalized with acute gastroenteritis in southern Ghana prior to the introduction of rotavirus vaccination.

## Results

During the period August 2007 to February 2011, 2277 stool specimens were tested by EIA of which 1099 (48.2%) were positive for rotavirus. Of the 1099 EIA positive specimens 977 (89%) were PAGE positive and 876 (89.7%) had sufficient material for further characterization by RT-PCR. Of the 876 specimens 741 (84.6%) and 709 (80.9%) were assigned a G and P genotypes respectively while 624 (71.2%) were assigned both G and P genotype.

### Genotypes

We identified eight G genotypes (G1, G2, G3, G4, G8, G9, G10 and G12). The most prevalent G genotypes G1, G2 and G3 accounted for 377 (50.9%), 139 (18.8%) and 95 (12.8%) of cases respectively. The 3 P genotypes (P[4], P[6] and P[8]) identified among the 709 specimens accounted for 9%, 30.7% and 36.1% of cases respectively. Mixed G (7.3%) and P (24.2%) genotypes were not uncommon. Non-typeable rotaviruses comprised 135/876 (15.4%) and 167/876 (19.1%) for G and P genotypes respectively. Table 1 shows the genotype combinations of circulating rotavirus strains.

**Table 1:** Rotavirus genotypes in young children hospitalized with severe acute gastroenteritis in southern Ghana: 2007 – 2011

	**P type**					**G type**	
	**P[4]**	**P[6]**	**P[8]**	**Pmix**	**P uncharacterized**	**Total**	**% Prevalence**
G1	15	23	177	99	63	377	43.0
G2	32	45	16	32	14	139	15.9
G3	1	56	7	5	26	95	10.8
G4	4	5	0	3	1	13	1.5
G8	1	0	0	0	0	1	0.1
G9	0	14	6	4	7	31	3.5
G10	1	23	0	2	1	27	3.1
G12	0	1	3	0	0	4	0.5
G mix	2	16	19	12	5	54	6.2
G uncharacterized	7	35	28	15	50	135	15.4
Total	63	218	256	172	167	876	100.0

**Figure 1:**
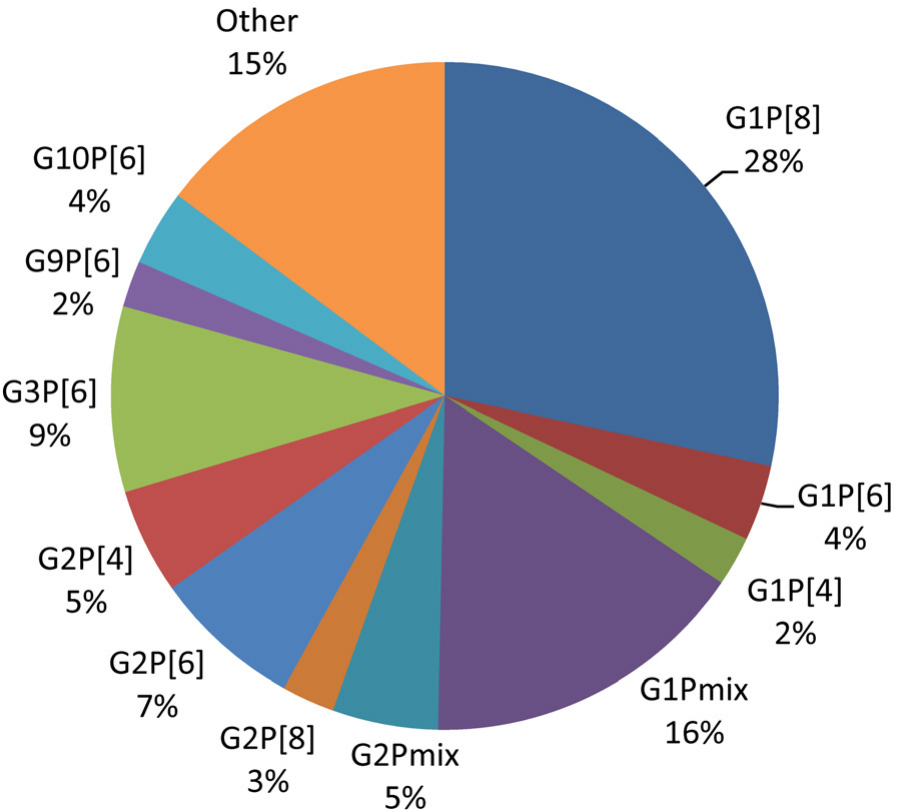
Frequencies (%) of rotavirus G/P combinations among 624 children hospitalized with acute gastroenteritis: 2007-2011.

Of the 624 specimens that were assigned both G and P genotypes 430 (68.9%) were single G and P genotype strains. Figure 1 shows the genotype distribution of all characterized strains; G1P[8], G1Pmix, G3P[6], and G2P[6] were the most prevalent strains and caused 60% of severe gastroenteritis cases in southern Ghana prior to the introduction of rotavirus vaccination.

### Temporal variation

The G1 genotype was the most prevalent cause of severe diarrhoea accounting for 35% – 50% of all acute gastroenteritis hospitalizations for each year of study. The prevalence of G2 genotype consistently decreased from 28% in 2008 to 2% in 2010 while G3 increased from 9% to 17% during the same period. There was only one case of G8 during the study period, but we also identified five cases of G1, 8 mixed genotypes.

**Figure 2:**
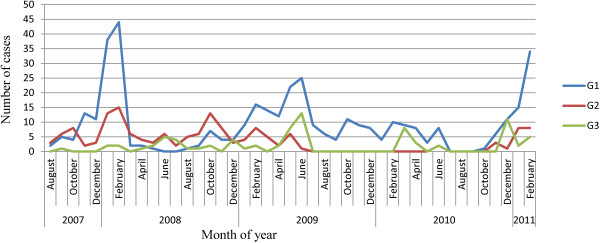
Seasonality of rotavirus G-genotypes in young children hospitalized with acute gastroenteritis in southern Ghana: 2007-2011.

The peak season for acute gastroenteritis was the dry cool months (December – February) in 2008 and 2010 and the rainy season (May – July) in 2009. The G1 genotype was the most prevalent genotype during the peak diarrhoeal seasons as shown in Figure 2. G1 and G3 occurred all year round while G2 prevalence fluctuated without a characteristic seasonal pattern. Of the 12 G4 genotypes identified in 2008, 7 occurred during the rainy season and 5 during the hot dry months (October and November). For G9 genotype, 11 of the 13 cases identified in 2008 occurred in the rainy season, there was no seasonal pattern in 2009. On the other hand G10 showed a predilection for the rainy season, 4/6 and 16/20 cases in 2008 and 2009 respectively occurred during the rainy season. There was only one case of G10 in 2010.

## Discussion

This report documents the genetic characterization of group A rotaviruses associated with severe acute gastroenteritis in children less than 5 years of age prior to the introduction of rotavirus vaccination in southern Ghana. G1P[8], G3P[6] and G2P[6] were the most prevalent strains and accounted for over 44% of all characterized strains and 65% of single strains of rotavirus identified in this cohort. Earlier reports from Ghana identified G2P[6], G3P[4] and G9P[8] as the most prevalent strains in the rural settings of the dry savannah northern part of the country [14]. The molecular epidemiology of rotavirus in this study showed great diversity in genotype combinations as reported from other African populations and in contrast to data from developed countries [7,15].

The G1 genotype was the most common cause of severe rotavirus gastroenteritis in this study as in other populations [7,16]. G1P[8] caused 28.4% of the infections in this Ghanaian cohort as compared to over 70% of cases in developed countries (United States, Europe, and Australia), 30% in South America and 23% in Africa [7]. Gentsch et al. reported that G1P[8], G2P[4], G3P[8], G4P[8], and G9P[8] represent 74% of globally identified strains [17]. However, these strains have been shown to be less prevalent in Africa comprising of only 37% of known strains [8], and 35.6% in this study. We did not identify any G4P[8] strain and G3P[8] and G9P[8] were rare accounting for 2% of the total cohort.

There was only one case of G8 genotype and five G1,8 mixed genotypes during the 43 months of this study. The prevalence of G8 in southern Ghana is quite low compared to what has been reported from other African countries [7,18,19]. Also G3 strains were mostly in combination with P[6] than P[8] in contrast to reports from elsewhere [20]. The novel combinations of G9P[6] and G9P[8] were the most common genotype combinations of G9 strain as observed in other regions.

We observed significant temporal and seasonal variations in the prevalence of some genotypes. G1P[8] was virtually non-existent during the rainy season (May – July) of 2008 and 2010 but it caused most of the gastroenteritis cases during the peak diarrheal season of 2009 which occurred in the rainy season. Other investigators have reported similar variable seasonal patterns of rotavirus disease in tropical countries [21,22]. It is thought to result from the genetic drifts of neutralizing antigens and reassortment of similar genes among locally co-circulating strains [20].

None of the genotypes exhibited a distinct seasonal pattern. Even though G10 occurred mostly in the rainy season, the surveillance period of this study is too short and long-term observations are required to evaluate this pattern. G10 has been shown to be more commonly associated with infections in animals [23] as such we hypothesize that the rainy season could create the right environment for co-infections with animal strains in young children living in the southern regions of Ghana. However, a study in northern Ghana found that G10 strains from Ghana were more closely related to human G10 strains than to animal G10 strains [24]. More studies are needed on the G10 strains from southern Ghana as the socio-environmental conditions in the south differ from those of the north.

The most prevalent genotype G1 caused severe diarrhoea in 47.5% of children under the age of 5 months; the age group targeted for rotavirus immunization. The great diversity of rotavirus strains observed in this study especially in young infants raises concerns about whether the vaccine strains G1P[8] would evoke sufficient heterotypic protection against other strains not present in the vaccine. Cross-protection with natural infection and vaccine-induced immunity has been reported [25-27]. However, as rotavirus vaccination is introduced into the routine immunization program it is important to closely monitor vaccine performance against heterotypic strains in Ghana.

The high proportion of mixed and non-typeable G and P types in this cohort is a limitation of this study. However, similar observations have been reported from other regions [28,29]. New molecular technologies are currently available for better characterization of rotaviruses. These facilities are currently not available in our research laboratory and were not provided for by sponsors of this surveillance study. Further studies on the non-typable specimens are recommended.

## Conclusion

There was great diversity of rotavirus strains associated with severe acute gastroenteritis in young children before the introduction of rotavirus vaccination in Ghana. Continuation of strain surveillance after the introduction of vaccination is recommended for evaluating the impact of vaccination on strain distribution and assessing effectiveness or otherwise of the rotavirus vaccine in use in Ghana.

## Methods

This study was conducted as part of the World Health Organization sponsored rotavirus surveillance study in selected African countries. The study was submiited to the Ethical and Protocol Review Committee of the University of Ghana Medical School. The risk to participants was considered as negligible (the inconvenience of filling a simple form ans scooping the stool of their hospitalized child in the specimen bottle provided). Children aged 0 – 59 months who were hospitalized for more than 24 hours with a primary diagnosis of acute gastroenteritis were eligible to be included in the study if the parents gave consent. Children with bloody stools or those whose parents did not give consent were excluded. Stool specimens collected from August 2007 to February 2011 were tested for rotavirus antigen at the University of Ghana Medical School Virology laboratory in Accra. Rotavirus positive specimens were subsequently sent to the Noguchi Memorial Institute of Medical Research rotavirus reference laboratory in Accra for determination of rotavirus genotype by polyacrylamide gel electrophoresis (PAGE) and reverse-transcriptase polymerase chain reaction (RT-PCR)*.* EIA positive stool specimens were stored at −20°C until genotype analysis was performed.

### Laboratory analysis

Rotavirus group A enzyme immuno-assay (IDEIA^TM^ kit, DAKO Diagnostics, United Kingdom) was performed on a 10% suspension of fecal specimen in a phosphate buffer saline as described by Asmah et al. [30]. Positive stool specimens were further subjected to PAGE. In some instances, all negative and positive samples were subjected to PAGE to screen for any non-group A rotaviruses. The viral dsRNA was extracted and purified from the 10% fecal suspension by the phenol/chloroform method as described by Steele et al. [31] and purified with an RNaid® Kit (Bio 101, Carlsbad, USA). The dsRNA was then electrophoresed for 20 hours at 100 V in a 10% vertical polyacrylamide slab gel. The RNA was visualized by the silver staining technique as described by Herring et al. [32].

All PAGE-positive and EIA-positive but PAGE-negative specimens were further characterized by RT-PCR as described by Armah et al. [33]. Briefly, a semi-nested multiplex RT-PCR was performed on purified dsRNA extracted from EIA and PAGE positive specimens after priming with VP7 and VP4 consensus primer pairs. A second round multiplex PCR was done for G-typing and P-typing by using specific primers [34,35]. All PCR products were also examined by gel electrophoresis in 1.2% agarose gel containing 4 μg/ml ethidium bromide and the G and P types were determined by the molecular weight of the amplicons.

### Data analysis

Laboratory data was entered into a designated register. All data were subsequently entered into a database (Access 2007 for Windows, Microsoft Corporation, USA) and analyzed using Stata version 10 (StataCorp, College Station, TX, USA).

## Competing interests

All the authors declare that they have no competing financial or non-financial interests. Though WHO provided support for this study authors received no funding for the work or preparation of the manuscript.

## Authors’ contributions

CEL and GEA participated in the design of the study. KWS carried out the immunoassays. SD and BL carried out the molecular genetic studies and participated in the analysis of the data. CEL drafted the manuscript in coordination with GEA and KWS. All authors read and approved the final manuscript.

## Authors’ information

CEL (MRCPCH) is the paediatrician in charge of rotavirus surveillance in the 3 hospitals where the data for this study was collected. KWS (PHD) is the head of the medical virology laboratory. GEA (PHD) is the head of the electron microscopy laboratory and regional rotavirus surveillance reference laboratory. SD (MSc) is the chief laboratory scientist and BL (BSc) is a laboratory scientist at the electron microscopy laboratory and regional rotavirus surveillance reference laboratory.
